# Predicting Response to Immunotherapy in Metastatic Renal Cell Carcinoma

**DOI:** 10.3390/cancers12092662

**Published:** 2020-09-18

**Authors:** Matthew D. Tucker, Brian I. Rini

**Affiliations:** Department of Medicine, Division of Hematology and Oncology, Vanderbilt University Medical Center, Nashville, TN 37232, USA; matthew.tucker@vumc.org

**Keywords:** biomarkers, immunotherapy, renal cell carcinoma, PD-L1

## Abstract

**Simple Summary:**

Immunotherapy-based treatment options have become standard of care in metastatic renal cell carcinoma. Despite significant improvement in overall survival with these therapies, the tumors of many patients will eventually progress. This review highlights the ongoing efforts to develop biomarkers to help predict which patients are most likely to benefit from treatment with immunotherapy.

**Abstract:**

Immunotherapy-based combinations, driven by PD-1, PD-L1, and CTLA-4 inhibitors, has altered the treatment landscape for metastatic renal cell carcinoma (RCC). Despite significant improvements in clinical outcomes, many patients do not experience deep or lasting benefits. Recent efforts to determine which patients are most likely to benefit from immunotherapy and immunotherapy-based combinations have shown promise but have not yet affected clinical practice. PD-L1 expression via immunohistochemistry (IHC) has shown promise in a few clinical trials, although variations in the IHC assays as well as the use of different values for positivity presents unique challenges for this potential biomarker. Several other candidate biomarkers were investigated including tumor mutational burden, gene expression signatures, single gene mutations, human endogenous retroviruses, the gastrointestinal microbiome, and peripheral blood laboratory markers. While individually these biomarkers have yet to explain the heterogeneity of treatment response to immunotherapy, using aggregate information from these biomarkers may inform clinically useful predictive biomarkers.

## 1. Introduction

An estimated 400,000 new renal cancers are diagnosed annually world-wide leading to over 175,000 deaths [1]. Early systemic therapies designed to target the immunogenicity of metastatic renal cell carcinoma (mRCC), such as interferon-alpha and high-dose IL-2, were effective in only a small percentage of patients [2,3]. While subsequent therapies designed against angiogenesis including tyrosine kinase inhibitors (TKI) targeting vascular endothelial growth factor (VEGF) and its receptor (VEGFR) improved response rates and progression-free survival, nearly all patients developed resistance [4].

The implementation of monoclonal antibodies against the immune checkpoint proteins programmed cell death 1 (PD-1), programmed death-ligand 1 (PD-L1), and anti-cytotoxic T-lymphocyte-associated protein-4 (CTLA-4) has dramatically changed the treatment paradigm for mRCC [5]. After demonstrating improved overall survival (OS) compared to the mammalian target of rapamycin (mTOR) inhibitor everolimus in the post-VEGF-R inhibitor setting, nivolumab (anti PD-1) became the first immune checkpoint inhibitor to gain FDA approval for advanced RCC in November of 2015 [6,7]. Subsequently, in April 2018, the immunotherapy combination nivolumab plus ipilimumab (anti-CTLA-4) gained approval in the first-line setting after demonstrating improved OS versus sunitinib [8]. In April and May of 2019, two additional immunotherapy-based combinations were approved in the first-line setting: pembrolizumab (anti-PD-1) plus the anti-VEGFR agent axitinib and avelumab (anti-PD-L1) plus axitinib [9,10,11]. Despite these advances, only a minority of patients treated with immunotherapy will have a durable response, prompting the search for predictive biomarkers. Since the early phases of development of immunotherapy in mRCC, tremendous efforts have been made towards understanding the biology of the tumor microenvironment (TME) to help identify candidate biomarkers, such as immunohistochemistry (IHC) expression of PD-L1, tumor mutational burden (TMB), polybromo-1 gene (*PBRM1*) mutations, human endogenous retroviruses (hERVs), gastrointestinal microbiota, sarcomatoid histology, and the neutrophil to lymphocyte ratio (NLR). 

## 2. Programmed Death-Ligand 1

Expression of PD-L1 (historically denoted as B7 homolog 1) on tumor cells and tumor-infiltrating lymphocytes was initially shown to be a poor prognostic marker for patients with renal cell carcinoma based on IHC analyses performed in 2004 [12,13]. Furthermore, in 2006 Thompson et al. performed a retrospective analysis of over 300 patients with mRCC and found that the 5-year cancer-specific survival rate was 42% for patients expressing PD-L1 versus 83% for patients who were negative [14]. Subsequently, a post-hoc analysis of the phase III trial COMPARZ, comparing efficacy of pazopanib to sunitinib, found that patients treated with either agent had significantly worse OS and progression-free survival (PFS) if they were PD-L1+ compared to those who were PD-L1− [15]. Thus, tumor PD-L1 expression is a negative prognostic factor in RCC and predicts against response to anti-VEGFR therapy.

Early phase clinical trials with anti-PD-1 monotherapy showed potential for the use of PD-L1 expression as a predictive biomarker for immunotherapy in mRCC [16,17]. The phase III clinical trial CheckMate 025 demonstrated improved efficacy of nivolumab over everolimus regardless of PD-L1 status, i.e., the marker was prognostic but not predictive [6]. Interestingly, the prior association of worse prognosis was observed in both groups, as patients who were PD-L1+ had numerically lower OS compared with PD-L1- patients. PD-L1 expression has been evaluated in several randomized clinical trials (Table 1).

In the phase III CheckMate 214 trial, evaluating nivolumab in combination with ipilimumab versus sunitinib, 91% (1002/1096) of patients in the intention-to-treat (ITT) population had quantifiable tumor tissue available for PD-L1 testing [8]. Tumors were positive if they had tumor cells (from baseline tumor samples prior to therapy) with > 1% PD-L1 expression as assessed using the Dako PD-L1 IHC 28-8 pharmDx test. Multivariate analysis of baseline factors was presented in the 32-month extended follow-up report [18] and showed that PD-L1 expression was a negative predictor for survival among patients treated with sunitinib [hazard ratio (HR) 0.70; 95% CI 0.52-0.93 for patients negative for PD-L1 expression]. However, PD-L1 was not associated with survival among patients treated with nivolumab plus ipilimumab even in univariate analysis, suggesting that combination immunotherapy was able to overcome the negative prognostic effects associated with PD-L1 expression.

**Table 1:** Clinical outcomes by PD-L1 expression status from phase III clinical trials of immunotherapy in mRCC.

Trial Name	Treatment + Arms	mOS PD-L1+	mOS PD-L1-	mOS ITT	mPFS PD-L1+	mPFS PD-L1-	mPFS ITT	CR% in PD-L1+	CR% in PD-L1-	ORR in PD-L1+	ORR in PD-L1-
CheckMate 214 [8]	Nivolumab + ipilimumab vs. sunitinib (intermediate and poor-risk)	NR vs. 19.6 mo (HR 0.45)	NR vs. NR (HR 0.73)	NR vs. 26.0 mo (HR 0.63)	22.8 mo vs. 5.9 mo (HR 0.46)	11.0 mo vs. 10.4 mo (1.00 *)	11.6 mo vs. 8.4 mo (HR 0.82 *)	16% vs. 1%	7% vs. 1%	58% vs. 22%	37% vs. 28%
KEYNOTE-426 [9,10]	Pembrolizumab + axitinib vs. sunitinib	HR 0.54 (12-mo OS)	HR 0.59 * (12-mo OS)	NR vs. 35.7 (HR 0.68) [10]	15.3 mo vs. 8.9 mo (HR 0.62)	15.0 mo vs. 12.5 mo (HR 0.87 *)	15.4 mo vs. 11.1 mo (HR 0.71) [10]				
JAVELIN Renal 101 [19]	Avelumab + axitinib vs. sunitinib	HR 0.83 *		HR 0.80 *	13.8 mo vs. 7.0 mo (HR 0.62)		13.3 mo vs. 8.0 mo (HR 0.69)	5.6% vs. 2.4%	1% vs. 1%	55.9% vs. 27.2%	47.1% vs. 27.3%
IMmotion151 [20]	Atezolizumab + bevacizumab vs. sunitinib	HR 0.84		HR 0.93 *	11.2 mo vs. 7.7 mo (HR 0.74)	11.2 mo vs. 9.5 mo (HR 0.89 *)		9% vs. 4%	3% vs. 1%	43% vs. 35%	33% vs. 32%
CheckMate 025 [6]	Nivolumab vs. everolimus	21.8 mo vs. 18.8 mo (HR 0.79 *)	27.4 mo vs. 21.2 mo (HR 0.77)				4.6 mo vs. 4.4 mo (HR 0.88 *)				

* reported HR is not statistically significant

Among patients with International Metastatic RCC Database Consortium (IMDC) favorable-risk disease, only 11% (11/115) of those treated with nivolumab plus ipilimumab and 12% (13/111) of those treated with sunitinib were PD-L1+ compared to 26% (100/384) and 29% (114/392) of patients with IMDC intermediate- and poor-risk disease [8]. Exploratory analysis according to PD-L1 expression was performed in the intermediate- and poor-risk patient population. The median PFS for PD-L1+ patients was 22.8 months with nivolumab plus ipilimumab versus 5.9 months with sunitinib (HR 0.46; 95% CI, 0.31–0.67), while the median PFS between nivolumab plus ipilimumab versus sunitinib was not significantly different among those who were PD-1L− (HR 1.00; 95% CI, 0.80–1.26). While overall survival was significantly longer with nivolumab plus ipilimumab versus sunitinib in both the PD-L1+ and negative groups, the degree of improvement in overall survival was greater in the PD-L1+ patients: HR 0.45 (95% CI, 0.29–0.71) in the PD-L1+ group and HR 0.73 (95% CI, 0.56–0.96) in the PD-L1− population. Additionally, the difference in ORR between nivolumab plus ipilimumab versus sunitinib was numerically higher in the PD-L1+ group with ORR of 58% with nivolumab plus ipilimumab versus 22% with sunitinib (*p* < 0.001), compared with 37% versus 28% (*p* = 0.03) in the PD-L1− group. Complete responses were also more frequent in the PD-L1+ group with 16% CR with nivolumab plus ipilimumab versus 1% with sunitinib, compared to 7% and 1% among respective PD-L1− patients. Thus, PD-L1 expression enriches for clinical benefit with combination nivolumab plus ipilimumab but cannot be used as a predictive biomarker given the significant benefit observed in the PD-L1− group.

The role of PD-L1 expression has also been explored in combinations of anti-VEGF therapy with immunotherapy. IMmotion150, a randomized phase II trial, investigated the clinical activity of atezolizumab with or without bevacizumab against sunitinib in patients with treatment-naive mRCC [21]. This trial included numerous ancillary biomarker investigations, including PD-L1 expression. Co-primary end points were PFS in both the ITT and in the PD-L1+ patient populations. PD-L1 was measured using the Ventana SP142 IHC assay, and PD-L1 was considered positive if >1% tumor-infiltrating immune cells (ICs) expressed PD-L1. The percentage of patients considered PD-L1+ among the three treatment groups were 59% sunitinib, 52% atezolizumab, and 50% atezolizumab + bevacizumab. The initial stratification was based on PD-L1 status of >5% (instead of >1%), which is thought to explain some of the imbalance among the treatment arms. The median PFS in the PD-L1+ population was 14.7 months with atezolizumab + bevacizumab versus 7.8 months with sunitinib (HR 0.64; 95% CI, 0.38–1.08), while the median PFS in the ITT population was 11.7 months with atezolizumab + bevacizumab versus 8.4 months with sunitinib (HR 1.00; 95% CI, 0.69–1.45). Furthermore, the hazard ratios for improvement in PFS were numerically improved with increasing levels of PD-L1 expression among patients treated with atezolizumab plus bevacizumab. Thus, PD-L1 expression enriched for response to this combination, although the overall activity of this regimen is lower compared to other immunotherapy-based doublets in mRCC.

The randomized phase III trial, IMmotion 151 further explored these findings. IMmotion151 enrolled patients with clear cell or sarcomatoid histology randomized to atezolizumab plus bevacizumab versus sunitinib [20]. Co-primary end points included investigator assessed progression-free survival in PD-L1+ patients and overall survival in the intention-to-treat population. PD-L1 was measured using the Ventana SP142 IHC assay, and PD-L1 was considered positive if >1% tumor-infiltrating immune cells (ICs) expressed PD-L1. Among patients in the PD-L1+ subset, median PFS was 11.2 months in the atezolizumab plus bevacizumab arm compared with 7.7 months in the sunitinib arm; HR 0.74 (95 CI 0.57–0.96; *p* = 0.0217). Similar to IMmotion 150, the HRs for PFS were numerically improved with increasing levels of PD-L1 expression. However, overall survival in the ITT population did not cross the prespecified significance boundary, with median overall survival HR 0.93 (0.76–1.14; *p* = 0.4751) at interim analysis. The HR for median overall survival in the PD-L1+ patients was 0.84 (0.62–1.15; *p* = 0.2857). ORR was 43% (76/178) in the PD-L1+ atezolizumab plus bevacizumab group compared to 35% (64/184) in the PD-L1+ sunitinib group, per investigator assessment. This difference was not seen among the PD-L1- group; 33% (90/276) for atezolizumab plus bevacizumab versus 32% (89/276) for sunitinib. For investigator assessed PD-L1+ patients, CR was 9% with atezolizumab plus bevacizumab vs. 4% with sunitinib, while CR was only 3% and 1% in the respective PD-L1− groups.

In the phase III KEYNOTE-426 trial [9], in which the combination pembrolizumab plus axitinib was compared to sunitinib, PD-L1 expression was not incorporated into the primary endpoint; however, PD-L1 expression was tested and reported in the exploratory analysis. Expression was assessed using the PD-L1 IHC 22C3 pharmDx assay (Agilent Technologies) and was calculated using the combined positive score [CPS; calculated as the number of PD-L1+ cells (tumor cells, lymphocytes, and macrophages) divided by the total number of tumor cells, multiplied by 100]. Seventy-seven percent of patients (822/1062) had tumor samples evaluable for PD-L1 expression, and of these 60.5% had a combined positive score >1. The 12-month OS rates among PD-L1+ patients were 90.1% with pembrolizumab plus axitinib and 78.4% with sunitinib (HR 0.54, 95% CI, 0.34–0.84). In the PD-L1− group the 12-month OS rates were 91.5% versus 78.3% respectively (HR 0.59, 95% CI 0.34–1.02). The median PFS among PD-L1+ patients was 15.3 months with pembrolizumab plus axitinib versus 8.9 months with sunitinib (HR 0.62, 95% CI 0.47–0.80), and in the PD-L1- group median PFS was 15.0 months versus 12.5 months (HR 0.87, 95% CI 0.62–1.23). Given marked benefit in both PD-L1+ and PD-L1− patients over sunitinib, there was no signal for use of PD-L1 expression as a predictive biomarker for treatment with pembrolizumab plus axitinib. However, it is notable that the poor prognostic association of PD-L1 expression with sunitinib was not observed in this study as had been shown previously, with 12 months OS 78.4% in the PD-L1+ patients and 78.3% in those who were PD-L1−. This difference may be at least partly explained by the use of a different assay and different methodology, namely the combined positive score, for determining PD-L1 expression.

The phase III JAVELIN Renal 101 trial, evaluating the combination avelumab plus axitinib versus sunitinib, incorporated PD-L1 expression into the combined primary endpoints of PFS and OS among PD-L1+ patients [11]. Expression was considered positive if >1% of immune cells were positive within the sampled tumor area as assessed by the Ventana PD-L1 SP263 assay (Ventana Medical Systems). Similar to the KEYNOTE-426 trial, JAVELIN Renal 101 also had a large number (69%, 560/812) of patients with evaluable samples positive for PD-L1. With a median follow-up of at least 13 months, PD-L1+ patients had a median PFS of 13.8 months with avelumab plus axitinib versus 7.0 months with sunitinib (HR 0.62, 95% CI 0.49–0.77) compared with 13.3 months versus 8.0 months (HR 0.69, 95% CI, 0.57–0.83) in the ITT group [19]. The overall CR rate was 3.8% with the combination avelumab plus axitinib, and 15 of these 17 patients with CR were PD-L1+. The CR rate was only 2.0% overall in the sunitinib group. Interestingly, the majority of these patients (7/9) were also PD-L1+. A new analysis of JAVELIN Renal 101 reassessed PD-L1 expression using the percentage of tumor cell positivity and found only 27% (218/812) of patients had expression >1% [22], and by using this approach 92% (196/212) would have also been considered positive using the immune cell algorithm. While there was no difference in PFS among the avelumab plus axitinib group (HR 0.89; 95% CI 0.65–1.22), PFS was shorter among PD-L1+ patients in the sunitinib arm (HR 1.57; 95% CI 1.16–2.14). Increasing the expression cutoff to 5%, 10%, and 25% did not lead to statistical difference among the avelumab plus axitinib group. Similar to KEYNOTE-426, PD-L1 status alone does not appear to predict response to immunotherapy in combination with axitinib.

One of the biggest drawbacks for PD-L1 as a predictive biomarker, is the variety of available tests and different methodologies for determining positivity. New biomarker analysis from CheckMate 214 presented at ASCO 2020 compared the previously reported PD-L1 expression data as defined using tumor cell expression >1% to the combined positive score [23]. Most notably, the percentage of patients determined to be PD-L1+ increased from 23% (113/498) to 61% (298/487) in the nivolumab plus ipilimumab group and from 25% (125/494) to 60% (298/493) in the sunitinib group, comparable to KEYNOTE-426 and JAVELIN Renal 101. The combination nivolumab plus ipilimumab significantly improved OS compared to sunitinib in both PD-L1+ and PD-L1− patients regardless of which test was used. However, stratified overall survival within the nivolumab plus ipilimumab group by PD-L1 combined positive score was not reported. Therefore, it remains unclear at this time whether the enrichment for response seen with PD-L1 expression (as initially reported using >1% positive tumor cells) remains using the combined positive score with this immunotherapy combination.

## 3. Tumor Mutational Burden

Tumor mutational burden (TMB) has been theorized to predict response to immunotherapy given increased formation of neoantigens on the tumor surface which lead to enhanced immunogenicity [24]. In June of 2020, the FDA announced a tumor-agnostic approval for pembrolizumab in patients whose tumors harbor TMB > 10 mutations per megabase (muts/Mb) [25], though the utility of TMB for predicting response to immunotherapy in RCC remains unproven.

Genomic profiling on over 1600 tumor samples from a variety of solid tumor types was performed using the MSK-IMPACT assay to examine the association between TMB and response to immunotherapy [26,27]. Given the heterogeneity of TMB between different histologies [28], TMB was analyzed using pre-specified cutoff percentages within each histology. Using a binary cutoff of 20%, a significant improvement in OS was observed across the entire cohort; HR 0.061 (*p* = 1.3 × 10^−7^). Patients with RCC made up 9% (*N* = 151) of the cohort. When limiting the analysis to this subgroup, no significant difference was found in OS between the patients in the top 20% of TMB and those below (cutoff 5.9 muts/Mb), HR 0.569. Using a more stringent cutoff of 10% (7.9 muts/Mb) or a more inclusive cutoff of 30% (cutoff 4.9 muts/Mb), a difference in OS was still not found.

Numerous retrospective analyses in RCC evaluating TMB have since been conducted with little to no association found. Labriola et al. evaluated 34 patients with mRCC treated with immunotherapy (32 with nivolumab, 2 with nivolumab plus ipilimumab, and 1 with pembrolizumab) who underwent genomic profiling with the PGDx elio panel [29]. Patients were grouped as either progressive disease or disease control (defined as stable disease or partial response). There was no significant difference observed in the TMB score between the two groups (*p* = 0.7682), with a mean TMB of 3.01 muts/Mb among the progressive disease group versus 2.63 muts/Mb in the disease control group. There were three patients who had a TMB score > 10 muts/Mb; two had progressive disease and one was in the disease control group.

Wood et al. examined a cohort of 431 patients with melanoma, non-small cell lung cancer, and RCC who had publicly available whole exome sequencing [30]. They determined TMB status based on consensus calls in DNA variants. Overall survival data was available for 56 patients with RCC, 50 of whom had reported response data available (excluding combination immunotherapy). Separating patients into two binary groups: TMB high (defined as those exceeding the disease-matched 80th percentile) and TMB low, there was no significant difference in overall survival (*p* > 0.05). Using logistic regression to evaluate for response probability and TMB, they found that TMB was a partial predictor of response in melanoma and non-small cell lung cancer but they found no significant difference among patients with RCC (*p* = 0.894).

Dizman et al. evaluated 91 patients at the City of Hope Comprehensive Cancer Center with mRCC who had undergone genomic profiling with DNA whole exome sequencing and RNA next-generation sequencing using the GEM ExTra assay [31]. Only patients whose genomic profiling was performed prior to initiation of systemic treatment were included for analysis. One cohort of patients were treated with immunotherapy (*N* = 32) and the other with VEGF-TKI therapy (*N* = 43). Eleven patietns (34%) patients in the immunotherapy cohort were treated with first-line nivolumab plus ipilimumab, while the remaining patients were treated with nivolumab monotherapy in either the second- or third-line settings. Patients were defined as with clinical benefit if they achieved complete or partial response of any duration or stable disease for at least six months. Overall, the median TMB was low at 1.2 muts/Mb (range 0.03–4.0) and no significant difference was seen between patients with clinical benefit versus no clinical benefit in either the immunotherapy cohort (*p* = 0.82) or the VEGF-TKI cohort (*p* = 0.091).

Braun et al. performed extensive genomic analyses on tumor samples from patients enrolled on the randomized phase III CheckMate 025 trial, treated with nivolumab monotherapy or the mTOR inhibitor everolimus and on the phase II CheckMate 010 trial [32]. The data were combined with existing whole exome sequencing and RNA-seq data from CheckMate 009 [33]. Whole exome sequencing data was available for 261 patients treated with nivolumab and 193 patients treated with everolimus. Clinical benefit was defined as having complete or partial response or stable disease with tumor shrinkage and PFS of at least six months, and they calculated TMB as the calculated sum of all non-synonymous mutations in each sample. No differences were observed in the total mutation burden between the clinical benefit group (*N* = 78) and the no clinical benefit group (*N* = 95); *p* = 0.81.

Interestingly, Huang et al. performed analysis on available somatic mutation data and transcriptome profiles from patients with clear cell RCC in the TCGA cohort (*N* = 537) [34]. TMB was defined as the total number of variants divided by the whole length of exons, 38 million (including base substitutions, deletions, and insertions). Pearson correlation analysis was used to evaluate expression of PD-1, PD-L1, and CTLA-4 with TMB. While no significant association was found between TMB and CTLA-4 (*p* = 0.270) or PD-1 (*p* = 0.493), a significant negative correlation between TMB and PD-L1 expression was determined (R = −1.51 and *p* = 0.006). Analysis from CheckMate 214 presented at ASCO 2020, showed no difference in PFS or OS between high TMB and low TMB within either the nivolumab plus ipilimumab arm or in the sunitinib arm [23], and recently published data from JAVELIN Renal 101 showed that TMB did not differentiate PFS in either the avelumab plus axitinib group (HR 1.09; 95% CI 0.79–1.50) or in the sunitinib group (HR 0.79; 95% CI 0.60–1.05 [22]. Overall, despite recent tumor-agnostic FDA approval for immunotherapy in tumors with TMB > 10 muts/Mb [25], TMB does not appear to reliably predict response in mRCC.

## 4. RNA Gene Expression

Gene expression profiling using RNA sequencing was evaluated in several randomized control trials (Table 2). McDermott et al. conducted pre-specified exploratory genomic analysis of IMmotion 150, the phase 2 trial of atezolizumab + bevacizumab versus sunitinib, and atezolizumab monotherapy versus sunitinib [21]. Gene expression analysis was performed by generating whole transcriptome profiles for 263 patients using RNA sequencing, TruSeq RNA Access (Illumina). Gene expression signatures previously found to be associated with angiogenesis (*VEGFA, KDR, ESM1, PECAM1, ANGPTL4,* and *CD34*), immune activation (*CD8A, EOMES, PRF1, IFNG,* and *CD274*), and myeloid inflammation (*IL-6, CXCL1*, *CXCL2*, *CXCL3*, *CXCL8*, and *PTGS2*) were used to group patients into high and low expression categories for each signature, separated by the median expression score derived for each group [35,36,37,38,39,40,41]. They found that the Angio^High^ subgroup had increased vascular density as determined by CD31 IHC, and that the Teff^High^ subgroup was associated with increased expression of PD-L1 on immune cells by IHC and with increased CD8+ T-cell infiltration.

When evaluated within the sunitinib treatment arm, high angiogenesis gene expression was associated with improved overall response (46% in Angio^High^ versus 9% in Angio^Low^) and PFS (HR 0.31; 95% CI, 0.18–0.55). While there was no difference in PFS among patients within the Angio^High^ subgroup, whether evaluated between atezolizumab plus bevacizumab versus sunitinib or between atezolizumab monotherapy versus sunitinib, there was an improvement in PFS observed among patients in the Angio^Low^ subgroup who were treated with atezolizumab plus bevacizumab versus sunitinib (HR 0.59; 95% CI, 0.35–0.98).

Within the atezolizumab plus bevacizumab arm, high immune gene expression was associated with improved overall response (49% in Teff^High^ versus 16% in Teff^Low^) and PFS (HR 0.50; 95% CI, 0.30–0.86). Additionally, when evaluated across treatment arms, Teff^High^ was associated with longer PFS with atezolizumab plus bevacizumab versus sunitinib (HR 0.55; 95% CI, 0.32–0.95).

High expression of genes involved in myeloid inflammation was associated with reduced PFS within the atezolizumab monotherapy arm (HR 2.98; 95% CI, 1.68–5.29) but not within the sunitinib arm. To further investigate the impact of myeloid inflammation and response to immunotherapy, the investigators examined the subgroup of patients with Teff^High^ and Myeloid^High^ tumors. Within this subgroup, patients treated with atezolizumab plus bevacizumab showed improved PFS versus those treated with atezolizumab alone (HR 0.25; 95% CI, 0.10–0.60). Interestingly, among patients in the Teff^High^Myeloid^Low^ subgroup, no significant differences were seen between the atezolizumab plus bevacizumab arm and the atezolizumab monotherapy arm. Overall, while these findings require further validation, they suggest that the addition of anti-VEGF treatment to immunotherapy, may help mediate some of the immunosuppressive effects of myeloid inflammation and may provide further insight into the efficacy of other anti-VEGF plus immunotherapy combinations, such as pembrolizumab or avelumab in combination with axitinib. Additionally, they support that expression of angiogenesis genes increase tumor susceptibility to sunitinib and that immune gene expression is associated with response to immunotherapy.

These genomic profiles were further validated in the prospective randomized phase III clinical trial, IMmotion151 and presented at ESMO 2018 [42]. RNA sequencing was performed on 823 patients. Patients with Teff^High^ had improved PFS with atezolizumab plus bevacizumab compared to sunitinib (HR, 0.76; 95% CI 0.59–0.99). While Angio^High^ was associated with improved PFS within the sunitinib arm (HR, 0.59; CI 0.47–0.75), there was no significant difference in PFS across treatment arms. Notably, Angio^High^ expression was more prevalent among patients with favorable risk as compared with intermediate/poor-risk, and Teff^High^ was more frequent among patients in the intermediate/poor-risk group, providing a biologic correlate of the differential clinical effects observed in CheckMate 214.

RNA expression profiling was also prospectively evaluated in the phase III clinical trial JAVELIN Renal 101 [11,22,43]. Researchers created whole transcriptome profiles using RNA sequencing on 720 baseline tumor samples and developed a new gene expression signature, Renal 101 Immuno, derived from 26 genes involved in T-cell proliferation, natural killer cell activation, interferon gamma signaling, and others. Using this signature, patients treated with avelumab plus axitinib who had high levels of expression (at or above the median level of expression) had significantly longer PFS compared with patients with low levels of expression (HR, 0.60; 95% CI, 0.44–0.83). Evaluating this signature in an independent dataset from the phase 1b JAVELIN 100 trial [44], high expression was again associated with prolonged PFS (HR 0.36; 95% CI 0.16–0.81). Using the gene expression signatures from the previous IMmotion studies, they also showed that the Angio^High^ signature was again associated with improved PFS within the sunitinib arm but was not significantly different between the avelumab + axitinib arm versus the sunitinib arm. Among patients with Angio^Low^ gene expression, PFS was significantly longer in the avelumab + axitinib arm compared with sunitinib. These data reinforce data from IMmotion 150 that Angio^low^ patients have better outcome with an immunotherapy-based regimen versus sunitinib monotherapy.

The RNA expression profiles from IMmotion150 and JAVELIN Renal 101 were further examined in a pooled analysis of available data from CheckMate 009, 010, and 025 [32]; however, no associations between high expression of any gene signature and improved clinical benefit, PFS, or OS were observed. One potential explanation for this difference is that the majority of patients included were treated with nivolumab in the second-line setting after prior anti-VEGF therapy, whereas patients in both IMmotion studies and JAVELIN Renal 101 were treatment-naïve. A multicenter retrospective analysis of 86 patients with mRCC treated with immunotherapy evaluated both a large T-effector gene panel and a smaller 5-Gene panel (*FOXP3*, *CCR4*, *KLRK1*, *ITK*, and *TIGIT*) [45]. While there was no difference observed between high and low expression of the larger T-effector panel, there was a significant difference in the ORR between the cohort with high 5-Gene expression versus low [31% (14/45) versus 2% (1/41); *p* = 0.001].

Biomarker data from CheckMate 214 presented at ASCO 2020 also reported the breakdown of six different gene expression signatures [23]. Twenty percent (109/550) of patients in the nivolumab plus ipilimumab arm and 19% (104/546) of patients in the sunitinib arm had tumor tissue evaluable to perform RNA sequencing. While Angio^High^ score (as per IMmotion150) was significantly associated with improved PFS within the sunitinib arm, no other observed significant differences were observed between the remaining gene expression signatures. Of note, this was the first reported study to evaluate these signatures with the use of an anti-CTLA-4 agent in combination with anti-PD-1; additionally, the percentage of patients with tumor evaluable for testing was low. However, when dichotomizing patients to PFS < 18 months versus > 18 months, they found differences in several HALLMARK [46] gene signatures: TNFalpha signaling via NFkB, epithelial mesenchymal transition, and inflammatory response. Gene expression signatures have successfully defined several subtypes of RCC related to varying degrees of immune involvement and angiogenesis; however, these signatures require further prospective validation prior to clinical use as predictive biomarkers.

## 5. *Polybromo-1* Mutations

In addition to mutations in the *von Hippel-Lindau* (*VHL*) gene, the pathogenesis of clear cell RCC includes a several secondary mutations including in *Polybromo-1* (*PBRM-1*), which has recently been implicated as a potential biomarker for immunotherapy [47]. Whole exome sequencing was performed to analyze pre-treatment tumor samples from 35 patients with mRCC treated with nivolumab on the prospective open-label phase I study, CA209-009 [33]. Patients were grouped into three different response categories for analysis: clinical benefit (patients with complete or partial response along with patients with stable disease if they had any objective reduction in tumor size lasting at least six months), no clinical benefit (patients with progressive disease leading to treatment discontinuation within three months), and intermediate benefit (patients who did not fit into the clinical benefit or no clinical benefit categories).

Truncating or loss of function mutations in *PBRM1* were more frequent in the clinical benefit group (9/11) compared with the no clinical benefit group (3/13, *p* = 0.012) with an odds ratio for clinical benefit of 12.93 (95% CI 1.54–190.8). OS and PFS were both significantly improved in patients with *PBRM1* loss of function (*N* = 19) compared to those with *PBRM1* intact (*N* = 16); *p* = 0.0074 and *p* = 0.29 respectively. They also evaluated *PBRM1* loss of function in an additional 63 patients who were treated with anti-PD-1(L1) therapy either alone or in combination with anti-CTLA-4 therapy. Again, tumors from patients deriving clinical benefit were more likely to harbor loss of function mutations in *PBRM1* (17/27) compared to those with *PBRM1* intact (4/19, *p* = 0.0071) with an odds ratio for clinical benefit of 6.10 (95% CI 1.42–32.64).

Braun et al. sought to validate these findings using archival tumor tissue from patients treated with nivolumab or everolimus from the randomized phase III trial, CheckMate 025 [48]. Of note, archival specimens were obtained prior to any treatment (including anti-VEGF therapy.) *PBRM1* mutations were identified in 29% (55/189) treated with nivolumab and in 23% (45/193) of patients who received everolimus. Among those treated with nivolumab, *PBRM1* mutations were present in 39% (15/38) of responding patients (either complete or partial response) compared to 22% (16/74) of non-responding patients (odds ratio 2.34, *p* = 0.04). Overall survival (HR, 0.65; *p* = 0.03) and progression-free survival (HR, 0.067; *p* = 0.03) were both associated with the presence of *PBRM1* mutations. However, among those treated with everolimus there was no significant difference between responders who harbored *PBRM1* mutations (1/5) and non-responders who harbored *PBRM1* mutations (10/56; *p* = 0.64). There was no significant association of *PBRM1* mutation with OS (HR, 0.81; *p* = 0.27) or PFS (HR 0.83; *p* = 0.32) among those treated with everolimus.

The association of *PBRM1* mutations with anti-PD-1 therapy was further investigated using pooled data from patients treated with nivolumab in either CheckMate-009, CheckMate-010, or CheckMate-025 who underwent whole exome sequencing [32]. Collectively, there was a significant benefit in OS and PFS for patients harboring *PBRM1* mutations (*p* < 0.001 and *p* = 0.0056 respectively). They also evaluated the presence of *PBRM1* mutations along with the degree of T-cell infiltration present in the tumor. Using CD8 immunofluorescence on 153 tumor samples from nivolumab treated patients, they quantified the density of CD8+ cells in the tumor center and at the tumor margin. Tumors were classified as “immune excluded” if at least five-fold more CD8+ cells were present in the tumor margin compared to the tumor center, “immune desert” if they were not “excluded” but still had below the 25th percentile of CD8+ cells in the tumor center, and “immune infiltrate” if they were not “excluded” and had greater than the 25th percentile of CD8+ cells in the tumor center. The majority (73%) of samples were classified as “immune infiltrated.” While there was no significant association between the degree of tumor infiltration and clinical benefit, there was an association observed between the presence of *PBRM1* mutations and lower T-cell infiltration (*p* = 0.013). *PBRM1* mutations were detected in 47% of immune “deserts” and 29% of immune “excluded” tumors, but only 22% of immune “infiltrated’ tumors (*p* = 0.01 for non-infiltrated versus infiltrated tumors).

However, there are also data to suggest that *PBRM1* mutations are associated with an immunosuppressive and pro-angiogenesis tumor microenvironment. Using a murine model, Liu et al. observed that *PBRM1* inactivation was associated with a less immunogenic tumor microenvironment, which was validated using human gene expression data from the TCGA-KIRC dataset [49]. The IMmotion150 dataset [21] was also analyzed, showing that patients with *PBRM1* mutations had a significantly lower ORR with both atezolizumab monotherapy and atezolizumab plus bevacizumab. Gene expression data from IMmotion150, TCGA, and the International Cancer Genome Consortium showed increased angiogenesis signatures among patients with *PBRM1* mutations from all three cohorts [49]. Clinical data from mRCC patients with pancreatic metastases demonstrated *PBRM1* mutations were associated with improved response to anti-VEGF therapy, supporting that *PBRM1* mutant tumors may have a more angiogenic phenotype [50]. Additionally, biomarker data from CheckMate 214 presented at ASCO 2020 showed no significant difference in PFS or OS between *PBRM1* wild type or mutant within either the nivolumab plus ipilimumab arm or in the sunitinib arm [23]. Furthermore, analysis from JAVELIN Renal 101 showed no association of *PBRM1* with PFS in either treatment arm [22]. Given conflicting results from multiple analyses, *PBRM1* mutations are not ready for clinical use as a predictive biomarker and require further investigation to understand their role in the tumor microenvironment. Some of the discrepant results may be due to the different populations studied, such as treatment-naïve versus VEGF-refractory.

## 6. Human Endogenous Retroviruses

Human endogenous retroviruses (hERVs) represent a group of long terminal repeat retrotransposons that collectively make up about 8% of the human genome [51,52]. Despite being normally silenced in somatic tissue, their expression has been reported in multiple cancer types, including RCC [53]. Aberrant transcriptional activation of hERVs has been theorized to induce an antitumor immune response and up-regulation of immune checkpoint pathways, increasing sensitivity to immunotherapy [54].

Using a cohort of 24 mRCC patients, Panda et al. used RNA sequencing to investigate an association of hERV expression and response to single agent PD-1(L1) therapy [54]. The RNA level of *ERV3-2* was measured using real-time quantitative PCRs (RT-qPCRs) with two independent primers. Regardless of which primer was used, they found that *ERV3-2* expression was significantly higher in responders compared with non-responders. Patients were also classified as either *ERV3-2* high or low based on an optimal cutoff derived from the receiver operating characteristic curves. *ERV3-2* high patients were significantly more likely to respond and had significantly longer PFS.

Post-hoc analysis from patients treated with nivolumab on CheckMate 010 was performed using RT-qPCR on 99 formalin-fixed paraffin-embedded tissue (FFPE) pretreatment tumors to determine expression levels of pan-*ERVE4*, pan-*ERV3.2*, *hERV4700 GAG* or *ENV*, and the reference genes *18S* and *HPRT1* [55]. Patients were dichotomized as high or low expression using the 25th percentile as the cutoff. Using this cutoff, only *hERV4700 ENV* was significantly associated with PFS and response. PFS was 7.0 months among the high-*hERV4700 ENV* group versus 2.6 months among the low expression group (*p* = 0.010), and the ORR for high-*hERV4700 ENV* was 35.6% versus 12.5% (*p* = 0.036).

Using pooled data from CheckMate 009, CheckMate 010, and CheckMate 025, Braun et al. found that hERV expression determined using RNA sequencing correlated with expression obtained by RT-qPCR; however, the authors note that *ERV3-2* expression was not reliably inferred using this technique, which highlights a potential limitation of using RNA sequencing to measure hERV expression in FFPE tissue [32]. Despite this limitation, they did find two hERVs (*ERV2282* and *EVR3382*) that were weakly associated with response, PFS, and OS when using expression as a continuous variable; however, when divided into high and low expression (based on median expression of each hERV) these were no longer significant for both improved PFS and OS. hERV expression presents a relatively new candidate biomarker that requires further prospective validation as well as improved reproducibility of technique.

## 7. Gastrointestinal Microbiome

Recent studies have shown a link between the intestinal microbiome and cancer immunosurveillance, including a role in response to immunotherapy [56,57,58]. A small Japanese study evaluated 31 patients with mRCC treated with immunotherapy and retrospectively separated them by antibiotic use within 30 days of treatment or not [59]. The median PFS for patients treated with antibiotics (*N* = 5) was 2.8 months compared with 18.4 months (*p* < 0.001) in the group without antibiotics (*N* = 26). Subsequently, Lalani et al. performed a retrospective analysis on two cohorts to explore the association of antibiotic use and response to immunotherapy in mRCC: the first, a single center cohort of patients who received anti-PD-1(L1) therapy (*N* = 146), and the second, a trial-database from patients treated with interferon, anti-VEGF therapies, or mTOR inhibitors (*N* = 4144) [60]. Antibiotic use was defined as anytime from 8 weeks before the start of therapy through 4 weeks after initiation. In the anti-PD-1(L1) cohort, patients with antibiotic exposure (*N* = 31) had a significantly lower ORR (12.9% versus 34.8%, *p* = 0.026) and shorter PFS (HR, 1.96, 95% CI 1.20–3.20, *p* = 0.007) compared to the group without antibiotic exposure (*N* = 115). In the trial-database cohort ORR was significantly lower (19.3% versus 24.2%, *p* = 0.005), PFS significantly shorter (HR 1.16, 95% CI 1.04–1.30), and OS significantly shorter (HR 1.25, 95% CI 1.10–1.41) in the antibiotic group compared with the no antibiotic group. However, in subgroup analysis the authors note that the difference in the trial-database group was driven by patients treated with interferon (*N* = 510) and prior cytokine therapy (*N* = 520), while no difference in OS was observed between antibiotic users in either the anti-VEGF (no prior cytokines) group (*N* = 2454) or in the mTOR group (*N* = 660).

Baseline fecal samples were collected from patients with mRCC treated with nivolumab on the NIVOREN GETUG-AFU 26 phase 2 clinical trial to investigate the relationship between the microbiome and response to immunotherapy [61]. Patients were dichotomized by prior antibiotic exposure, namely those who had received antibiotics within the two months prior to treatment with nivolumab (*N* = 11) and those without prior antibiotic exposure (*N* = 58). The ORR was lower in the antibiotic group at 9% versus 28% in the group without prior antibiotics (*p* < 0.03). Median PFS and OS were also longer in the no antibiotic group (5 months and NR) compared with the antibiotic group (2 months and 25 months; *p* = 0.03 and *p* = 0.04). They subsequently performed analysis of the relative taxonomic abundance for prevalent fecal bacteria between the two groups and found overrepresentation of *Eubacterium rectale* (*p* = 0.02) in the no antibiotic group and overrepresentation of *Erysipelotrichaceae bacterium* and *Clostridium hathewayi* in the antibiotic group (*p* < 0.02).

Additionally, they separated the group without prior antibiotic exposure into responders (*N* = 30) and non-responders (*N* = 28) and compared the various taxonomical fecal bacterial profiles. Patients in the responder group were more likely to harbor overrepresentation of *Akkermansia muciniphila, Bacteroides salyersiae*, and *Eubacterium siraeum* compared with non-responders. Likewise, patients in the non-responder group were more likely to have overrepresentation of *Erysipelotrichaceae bacterium, Clostridium hathewayi*, and *Clostridium clostridioforme*.

Recently, Salgia et al. performed an observational study of 31 patients with mRCC treated with either nivolumab monotherapy (77%) or nivolumab plus ipilimumab (23%) and assessed the gut microbiota composition using metagenomic sequencing [62]. Of note, patients with antibiotic exposure within 14 days were excluded. Using the Shannon diversity index, greater microbial diversity was associated with clinical benefit (*p* = 0.001). Prospective and controlled studies are warranted to further explore and validate using the microbiome to predict response to immunotherapy, as well as to explore the role of alteration of the host microbiota to enhance therapeutic response.

## 8. Sarcomatoid Differentiation

Sarcomatoid differentiation, present in around 10% of patients with RCC, is associated with more aggressive clinical features, including shorter time to relapse, worse IMDC prognostic classification, and shortened PFS and OS with anti-VEGF therapies [63,64,65].

A comparison of 40 tumors with sarcomatoid histology present versus clear cell RCC without sarcomatoid features found that tumors with sarcomatoid histology were significantly more likely to have co-expression of PD-L1 on tumor cells and on tumor-infiltrating-lymphocytes [66], suggesting the potential for improved response to immunotherapy. In the IMmotion151 trial, 61% (86/142) of patients with sarcomatoid histology were positive for PD-L1 expression [67], and analysis presented at ESMO 2018 showed that tumors with sarcomatoid histology were more likely to be Angio^Low^ and Teff^High^ compared with non-sarcomatoid tumors [42]. The HR for PFS in the sarcomatoid subgroup was 0.45 (95% CI 0.26–0.77) in the PD-L1+ population and 0.52 (0.34–0.79) in the ITT population [67]. Given that PFS was improved in both the PD-L1+ and PD-L1− populations, sarcomatoid histology may be an independent predictor of response.

Post-hoc analysis of patients from CheckMate 214 identified sarcomatoid features in 16.4% (139/847) of patients with intermediate/poor-risk RCC [68]. Overall survival was significantly improved in this subgroup with nivolumab plus ipilimumab versus sunitinib (HR 0.45; 95% CI 0.3–0.7). The ORR was 60.8% with nivolumab plus ipilimumab versus 23.1% with sunitinib (*p* < 0.0001), and the CR rate was 18.9% versus 3.1% respectively. Of note, 52% (69/133) of patients with quantifiable PD-L1 expression were positive. Iacovelli et al. performed a meta-analysis of the four published phase III randomized combination immunotherapy clinical trials that included patients with sarcomatoid histology [69]. Collectively there were 467 patients included with sarcomatoid features present, 226 randomized to immunotherapy-based combination treatment and 241 randomized to sunitinib. Overall, PFS significantly favored treatment with immunotherapy-based combinations (HR 0.56; 95% CI, 0.43–0.71). Excluding JAVELIN Renal 101 (given OS data was not mature at time of analysis), OS also significantly favored treatment with immunotherapy-based combinations (HR 0.56; 95% CI 0.43–0.82). The ORR was 52.6% for combination therapy versus 20.7% with sunitinib, with CR rates of 11.5% and 0.8% respectively. Overall, this meta-analysis supports the use of immunotherapy-based combinations in the front-line setting for patients with mRCC with sarcomatoid features, although if a specific regimen has greater effect awaits further study. Additional work exploring the role of immunotherapy-based combinations in other distinct RCC histologies is necessary.

## 9. Neutrophil to Lymphocyte Ratio

The baseline neutrophil to lymphocyte ratio (NLR) has been shown to have a negative prognostic association with RCC and to predict a more aggressive phenotype [70,71,72]. While the poor prognostic association of PD-L1 expression appears to be mitigated by immunotherapy [8,9,10], this does not appear to be the case for NLR.

Zahoor et al. performed a retrospective analysis of 90 patients treated with nivolumab as second-line or later therapy for mRCC. After multivariate analysis, a higher baseline NLR was associated with an increased risk of progression (HR 1.86, 95% CI, 1.05–3.29) [73]. Additionally, Lalani et al. performed a retrospective review of 142 patients treated with anti-PD-1(L1) therapies and found that baseline NLRs were significantly higher in the poor IMDC risk group compared to those with favorable or intermediate risk (*p* < 0.001) [74]. Interestingly, the results indicate that the NLR between 4 to 8 weeks post treatment initiation was a more accurate predictor of response than at baseline, and an increase in NLR > 25% was associated with shorter PFS (HR 2.60, 95% CI 1.53–4.39).

Furthermore, exploratory analysis of the JAVELIN Renal 101 trial, presented at ASCO 2020, demonstrated significantly improved OS (HR 0.51; 95% CI 0.30–0.87) and a trend toward improved PFS (HR 0.85; 95% CI 0.63–1.15) among patients treated on the avelumab plus axitinib arm who had a baseline NLR < the median compared to patients with baseline NLR > the median [75]. While the overall differences between groups is somewhat modest, the ratio is cost-effective and easily performed in clinic without the need for additional testing. Additional prospective validation is needed for pre-treatment laboratory biomarkers such as NLR.

## 10. Conclusions

While immunotherapy-based combinations have become the standard of care for first-line mRCC, the majority of patients will eventually progress on these regimens. Additionally, immune-related adverse events can lead to serious toxicity and have the potential to be life-threatening [76]. Therefore, the ability to predict which patients are most likely to respond would have significant clinical impact. Despite tremendous investigation on numerous candidate biomarkers, none have yet proven ready for clinical practice.

PD-L1 expression has been shown to enrich for response, most notably in CheckMate 214; however, patients with negative expression can still respond and maintain the potential for complete response. Furthermore, the commercially available anti-PD-L1 clones currently in use are highly variable, and PD-L1 expression patterns have been shown to be heterogenous throughout different tumor regions [77]. Therefore, immunotherapy should not be withheld for patients who are known to be PD-L1−. New imaging modalities are being developed to quantify PD-1 and PD-L1, which may help reduce some of the variability in future studies [78,79]. TMB, while recently approved as a tumor-agnostic biomarker for response to pembrolizumab, has been shown to be an unreliable predictor in RCC and should not be used in clinical decision making for these patients.

Gene expression using RNA sequencing has generated new understanding into the biology of RCC and patterns of response to therapy. Improvement in PFS with atezolizumab plus bevacizumab were observed in both IMmotion150 and IMotion151 among Teff^High^ patients; however, this improvement was not observed in updated analysis of CheckMate 214. Future prospective trials should incorporate pre-specified analyses of gene expression signatures to assess their potential for clinical utility.

Individual gene alterations, such as loss of function mutations in *PBRM1*, have demonstrated mixed results, and should not be used clinically at this time. Likewise, while antibiotic exposure has been associated with decreased response to immunotherapy, these data have yet to be prospectively validated and should neither prohibit patients who have recently required antibiotics from receiving immunotherapy nor should it limit clinicians’ use of antibiotics for infected patients. Given the enhanced efficacy observed among patients with sarcomatoid differentiation, these patients should receive upfront immunotherapy-based combinations instead of VEGF-TKI alone when systemic therapy is warranted.

While data regarding the NLR as a predictive biomarker is limited and is impacted by the known poor prognostic associations in RCC. However, such peripheral blood biomarkers have the benefit of being cost-effective and readily available; continued efforts toward identifying laboratory biomarkers is warranted.

In addition to developing biomarkers associated with radiographic response, there are several other facets of clinical practice that may be improved with the addition of biomarkers. Biomarkers that predict durability may help identify patients who can safely discontinue therapy after achieving a clinical response, and biomarkers that predict immune-related adverse events may help determine which patients should be observed more closely and potentially for which immunotherapy should be avoided or limited. While some candidate biomarkers may only enrich for response, these insights also help with developing novel therapies and combinations which may lead to improved outcomes with immunotherapy.

## Figures and Tables

**Table 2:** Gene expression signatures evaluated from randomized controlled trials in mRCC.

Biomarker	Trial	Patient Population	Key Findings
IMmotion 150 Angio Signature (*VEGFA*, *KDR*, *ESM1*, *PECAM1*, *ANGPTL4*, *CD34*)	CheckMate 214 [23]	Within sunitinib arm	Improved PFS among Angio^High^ (0.58)
	IMmotion150 [21]	Within sunitinib arm	ORR Angio^High^ 46% vs. Angio^Low^ 9%
		Atezolizumab + bevacizumab vs. sunitinib	Improved PFS among Angio^Low^ with atezolizumab + bevacizumab (HR 0.59)
	IMmotion151 [42]	Within sunitinib arm	Improved PFS among Angio^High^ (0.59)
		Atezolizumab + bevacizumab vs. sunitinib	Improved PFS among Angio^Low^ with atezolizumab + bevacizumab (HR 0.68)
	JAVELIN Renal 101 [22,43]	Within sunitinib arm	Improved PFS among Angio^High^ (0.64)
		Avelumab + axitinib vs. sunitinib	Improved PFS among Angio^Low^ with avelumab + axitinib
IMmotion 150 Teff Signature (*CD8A*, *EOMES*, *PRF1*, *IFNG*, *CD274*)	CheckMate 214 [23]	Within sunitinib arm	No difference in OS or PFS
		Within ipilimumab + nivolumab arm	No difference in OS or PFS
	IMmotion150 [21]	Within atezolizumab + bevacizumab	ORR T_eff_^High^ 49% vs. T_eff_^Low^16%
		Atezolizumab + bevacizumab vs. sunitinib	Improved PFS among Teff^high^ with atezolizumab + bevacizumab (HR 0.55)
	IMmotion151 [42]	Atezolizumab + bevacizumab vs. sunitinib	Improved PFS among Teff^high^ PFS with atezolizumab + bevacizumab (HR 0.76)
	JAVELIN Renal 101 [22]	Within avelumab + axitinib	Trend toward improved PFS among Teff^high^ (HR 0.79, 95% CI 0.58-1.08)
		Within sunitinib arm	No difference in PFS
IMmotion 150 Myeloid Signature (*IL6*, *CXCL1*, *CXCL2*, *CXCL3*, *CXCL8*, *PTGS2*)	CheckMate 214 [23]	Within sunitinib arm	No difference in OS or PFS
		Within ipilimumab + nivolumab arm	No difference in OS or PFS
	IMmotion150 [21]	Within atezolizumab arm	Reduced PFS among Myeloid^High^ (HR 2.98)
		Within atezolizumab + bevacizumab arm	Reduced PFS among Myeloid^High^ (HR 1.71)
		Atezolizumab vs. sunitinib	Reduced PFS among Myeloid^High^ with atezolizumab (HR 2.03)
		Atezolizumab + bevacizumab vs. sunitinib	No difference in PFS
	JAVELIN Renal 101 [22]	Within sunitinib arm	No difference in PFS
		Within avelumab + axitinib arm	No difference in PFS
IMmotion 150 Myeloid^high^ vs. Myeloid^low^ in Teff^high^	CheckMate 214 [23]	Within sunitinib arm	No difference in OS or PFS
		Within ipilimumab + nivolumab arm	No difference in OS or PFS
	IMmotion150 [21]	Within atezolizumab arm	Reduced PFS among Teff^High^Myeloid^High^ (HR 3.82)
		Atezolizumab vs. atezolizumab + bevacizumab	Improved PFS among Teff^High^Myeloid^High^ with atezolizumab + bevacizumab (HR 0.25)
	JAVELIN Renal 101 [22]	Within sunitinib arm	No difference in PFS
		Within avelumab + axitinib arm	No difference in PFS
JAVELIN Renal 101 Immuno (*CD3G*, *CD3E*, *CD8B*, *THEMIS*, *TRAT1*, *GRAP2*, *CD247*, *CD2*, *CD96*, *PRF1*, *CD6*, *IL7R*, *ITK*, *GPR18*, *EOMES*, *SIT1*, *NLRC3*, *CD244*, *KLRD1*, *SH2D1A*, *CCL5*, *XCL2*, *CST7*, *GFI1*, *KCNA3*, *PSTPIP1*)	CheckMate 214 [23]	Within sunitinib arm	No difference in OS or PFS
		Within ipilimumab + nivolumab arm	No difference in OS or PFS
	JAVELIN Renal 101 [22]	Within avelumab + axitinib	Improved PFS among Immuno^high^ with avelumab + axitinib (HR 0.60)
		Within sunitinib arm	No difference in OS or PFS
Tumor Inflammation Signature (*PSMB10*, *HLA-DQA1*, *HLA-DRB1*, *CMKLR1*, *HLA-E*, *NKG7*, *CD8A*, *CCL5*, *CXCL9*, *CD27, CXCR6*, *IDO1*, *STAT1*, *TIGIT*, *LAG3*, *CD274*, *PDCD1LG2*, *CD276*)	CheckMate 214 [23]	Within sunitinib arm	No difference in OS or PFS
		Within ipilimumab + nivolumab arm	No difference in OS or PFS
